# Problem-Solving Education to Prevent Depression Among Low-Income Mothers: A Path Mediation Analysis in a Randomized Clinical Trial

**DOI:** 10.1001/jamanetworkopen.2018.0334

**Published:** 2018-06-29

**Authors:** Michael Silverstein, Howard Cabral, Mark Hegel, Yaminette Diaz-Linhart, William Beardslee, Caroline J. Kistin, Emily Feinberg

**Affiliations:** 1 Department of Pediatrics, Boston Medical Center, Boston, Massachusetts; 2 Department of Pediatrics, Boston University School of Medicine, Boston, Massachusetts; 3 Department of Biostatistics, Boston University School of Public Health, Boston, Massachusetts; 4 Department of Psychiatry, Dartmouth-Hitchcock Medical Center, Lebanon, New Hampshire; 5 Heller School for Social Policy and Management, Brandeis University, Waltham, Massachusetts; 6 Department of Psychiatry, Boston Children’s Hospital, Harvard Medical School, Boston, Massachusetts

## Abstract

**Importance:**

Although problem solving has been an important component of successful depression prevention and treatment interventions, evidence to support problem solving’s mechanism of action is sparse.

**Objective:**

To understand the mechanism of an efficacious depression prevention intervention, problem-solving education (PSE).

**Design, Setting, and Participants:**

A multivariate path analysis was embedded within a randomized efficacy trial (February 15, 2011, to May 9, 2016). Participants were mothers with depressed mood, anhedonia, or depression history (but not in current major depressive episode) at 1 of 6 Head Start agencies. Participants were followed up for 12 months with serial assessments of potential intervention mediators and depressive symptoms.

**Interventions:**

Problem-solving education (n = 111) and usual Head Start services (n = 119).

**Main Outcomes and Measures:**

Primary outcomes were depressive symptom elevations, which were measured bimonthly. Eight plausible intervention mediators were assessed: problem-solving ability; mastery; self-esteem; perceived stress; behavioral activation; and avoidant, problem-focused, and social coping.

**Results:**

Among 230 participants, 152 (66.1%) were Hispanic; mean (SD) age was 31.4 (7.3) years. Based on associations with either PSE participation or depressive symptom outcomes, problem-solving ability, perceived stress, behavioral activation, and problem-focused coping were included in a parsimonious, multivariate path model. In this model, only perceived stress was associated with both PSE participation and depressive symptoms. Participants in the PSE group had adjusted standardized perceived stress change scores that were 11% lower than controls (95% CI, −0.19 to −0.03), and improvement in perceived stress generated an adjusted rate ratio (aRR) of 0.42 (95% CI, 0.33-0.53) for depressive symptom elevations. Participants in the intervention group also had standardized behavioral activation change scores 15% greater than controls (95% CI, 0.01-0.30) and problem-focused coping change scores 17% greater than controls (95% CI, 0.03-0.31); however, changes in these constructs were not associated with a differential rate of depressive symptom elevations. The direct effect of PSE on depressive symptom elevations (aRR, 0.72; 95% CI, 0.52-0.97) was greater than the mediated effect explained by improvement in perceived stress (aRR, 0.91; 95% CI, 0.85-0.98).

**Conclusions and Relevance:**

Problem-solving education is efficacious in preventing depressive symptoms and appears to work by decreasing perceived stress; however, the mechanism for much of PSE’s impact on depression remains unexplained. These results can be used to simplify the intervention model in preparation for effectiveness testing.

**Trial Registration:**

ClinicalTrials.gov Identifier: NCT01298804

## Key Points

**Question:**

What are the principal mechanisms by which problem-solving education prevents depression among low-income mothers?

**Findings:**

In this mediation analysis of 230 Head Start mothers participating in a randomized clinical trial, those receiving problem-solving education experienced a reduction in depressive symptom episodes. Across an array of plausible theory-based intervention mediators, improvement in perceived stress was associated with both intervention participation and depressive symptom outcomes; however, the mechanism for much of the intervention’s impact on depression remained unexplained.

**Meaning:**

Problem-solving interventions may reduce depressive symptom burden, in part, by helping recipients manage stress; successfully scaling such interventions may require expanding intervention components related to stress management and reducing components that are unrelated.

## Teaser

This study uses data from a clinical trial suggesting that problem-solving education (a form of cognitive behavioral therapy [CBT]) can prevent depression in low-income mothers to explore the mechanisms by which the intervention might be effective.

## Introduction

Maternal depression disproportionately affects low-income and minority women.^1^ These women face a variety of cultural and logistic barriers that impede engagement with mental health services, resulting in health disparities for both themselves and their children.^2,3^ Although much of the clinical research on maternal depression has focused on treatment, a growing body of work has shown that depression can be prevented.^4^ Thus, embedding maternal depression prevention strategies in accessible venues, outside traditional medical settings, is a potentially important public health strategy to reduce mental health disparities. In its 2009 report *Depression in Parents, Parenting, and Children,* the National Academy of Medicine endorsed this concept and called for interventions that take place in community-based, family-focused venues.^1^

Responding to this call, our research team recently completed a randomized efficacy trial of problem-solving education (PSE) in Head Start, a federally funded early learning program that provides services for approximately 1 million low-income families each year.^5^ Problem-solving education is a cognitive-behavioral depression prevention intervention, designed specifically for low-income mothers.^6^ Intervention models based on problem solving are well accepted in the fields of depression management and prevention^7,8,9^ and are based on the premise that because daily problems perpetuate depressive symptoms, working through these problems systematically can reduce symptoms and improve functioning.^10^

In our trial, Head Start mothers with subthreshold depressive symptoms who received PSE experienced a 40% reduction in the incident rate of clinically significant symptom episodes over a 12-month period, compared with treatment-as-usual controls.^11^ Although PSE is unique in that it was embedded as a lay-delivered program within a preschool setting, its intervention components are consistent with other successful problem-solving approaches.^7,12,13^ While evidence supporting the effectiveness of these approaches is solid,^7,8,9,14,15^ evidence that sheds light on problem solving’s mechanism of action is not as robust. To our knowledge, prior work suggests only that problem-solving models work by breaking down avoidant coping patterns.^16^

Understanding the mechanisms by which complex behavioral interventions work is important to developing effective intervention strategies that can be adapted to diverse venues and populations.^17^ By understanding such mechanisms, complex interventions can be distilled into ones that are simpler to implement, have greater potential to stand up to effectiveness testing, and make greater public health impact. For this reason, in the context of our randomized efficacy trial of PSE, we conducted a series of prespecified mediation analyses to understand PSE’s mechanism of action.

## Methods

### Design

We embedded a path mediation analysis within a parallel-group randomized efficacy trial. Details of our trial’s methods and its adherence to the Consolidated Standards of Reporting Trials (CONSORT) reporting guideline have been reported previously.^11^ The Boston University Medical Center institutional review board approved this study. All participants provided written informed consent. The full trial protocol is available in Supplement 1.

### Participants and Setting

We enrolled participants from February 15, 2011, to May 20, 2015. We worked in 6 Head Start preschool centers within a single metropolitan area serving families of children from birth to 5 years at or below the federal poverty level. We enrolled mothers at increased risk for depression, but excluded those in current major depressive episodes.^18^ *At risk* was defined as depressed mood or anhedonia according to the 2-question Patient Health Questionnaire-2,^19^ or a recent history of depression according to the Composite International Diagnostic Interview.^20^ A major depressive episode was determined by the Mini International Neuropsychiatric Interview.^21^ We excluded mothers with high levels of suicidal ideation according to the MacArthur Initiative on Depression and Primary Care’s suicide screen^22^ and with cognitive limitation according to the MacArthur Competence Assessment Tool.^23^ We enrolled English- and Spanish-speaking mothers.

### Randomization

We used stratified, blocked randomization, allocating participants 1:1 to PSE or usual Head Start services according to computer-generated lists. Randomization occurred independently at each Head Start site in strata defined by depression history and was balanced within randomly varying blocks of 2 and 4. Lists were concealed in opaque envelopes. Outcome assessors, investigators, and Head Start personnel were masked to study allocation.

### Study Groups

Sessions instructing participants in PSE were one-on-one, workbook-based interactions, adapted from Hegel and Areán’s problem-solving treatment manual.^24^ Sessions were delivered in the home or in the Head Start center; they typically lasted 30 to 60 minutes. Consistent with efficacy trial design, PSE providers were paid study personnel; none, however, had formal mental health training or a degree higher than a bachelor’s. A full course of PSE involved 6 sessions, delivered weekly or biweekly. Each session included 7 steps: defining a problem, goal setting, generating solutions, implementing decision-making guidelines, evaluating solutions, implementing solutions, and evaluating outcomes. The sessions placed equal emphasis on behavioral activation and the specification of a problem; this was designed to allow clients to focus on action planning and to provide a clear future orientation to the interaction. Providers of PSE instruction learned motivational interviewing techniques to promote intervention adherence.^25^

Usual Head Start services (which were available to mothers in both study groups) included family needs assessments, home visitation, parenting groups, referrals to behavioral health services, and assistance with accessing community resources for food, job training, and housing.

### Intervention Provider Training, Fidelity Monitoring, and Supervision

We trained 15 intervention providers. Training workshops lasted 1 to 2 days and were followed by up to 5 standardized training cases. Trainees were certified as PSE providers after completing 2 cases in which they met fidelity criteria according to a standardized checklist developed in prior work.^26^

Participants were randomly assigned to linguistically matched providers. We audiotaped 1 randomly selected session for each participant and used the same fidelity criteria as in provider training. Fidelity was rated according to the proportion of core PSE components delivered appropriately on a scale from poor (<60%) to excellent (≥90%). Over the course of the trial, all PSE providers met weekly with a master-level social worker (Y. D.-L.) to review caseloads, troubleshoot problems with intervention delivery, discuss clients who had difficulty engaging with the intervention, and reflect on the experience of working with clients with depressive symptoms and related vulnerabilities.

### Baseline Data

Prior to randomization, we collected mothers’ self-reported age and number of children, race and ethnicity, education level, work status, and single- vs dual-parent household status.

We assessed overall problem-solving ability with the Social Problem Solving Inventory, which measures problem orientation and problem-solving skills.^27,28,29^ We used the Pearlin Mastery Scale and Rosenberg Self-esteem Scale to assess mastery (the degree to which individuals perceive themselves as in control of their lives) and self-esteem, respectively.^30,31^ We assessed perceived stress with the Perceived Stress Scale, domains of which include unpredictability, lack of control, burden overload, and stressful circumstances.^32^

We assessed behavioral activation with the Behavioral Activation for Depression Scale.^33^ We assessed coping styles with the Brief COPE,^34^ which we operationalized into problem-focused, avoidant, and social coping subscales according to the classification system used by Oxman and colleagues.^16^

We assessed anxiety symptoms with the Beck Anxiety Inventory^35^ and trauma history and posttraumatic stress disorder (PTSD) symptoms with the Modified PTSD Symptom Scale.^36^ We assessed depressive symptoms with the Quick Inventory of Depressive Symptomatology (QIDS)^37^ and used the most widely accepted cut point of a score greater than or equal to 11, the clinical threshold for moderately severe symptoms.^38^

### Outcome Assessment

We followed participants for 12 months, beginning data collection 2 months after randomization. We assessed depressive symptoms bimonthly, operationalizing our primary outcome as elevations to the moderately severe threshold (QIDS ≥11).

### Mediating Factors

Acknowledging the evidence that problem-solving interventions can affect depression in the absence of measurable effects on problem-solving abilities,^7,39^ we identified problem-solving skills, mastery, self-esteem, and perceived stress as potential mediating constructs because they are consistent with PSE’s theoretical mechanism of action.^10^ Given PSE’s emphasis on goal setting and action planning, we also identified behavioral activation as a potential mediator. Because of prior evidence that suggests problem-solving interventions may work by reducing avoidant coping styles,^16^ we identified 3 key coping strategies as potential mediators: avoidant coping, problem-focused coping, and social coping. We assessed each of these potentially mediating constructs at 6 and 12 months following randomization, using the same instruments as at baseline.

### Statistical Analysis

To estimate intervention effect, we conducted intention-to-treat analyses using a set of binary variables to model the effects of Head Start site.^40^ We used negative binomial regression to compare rates of depressive symptom elevations over the follow-up period, using an offset to standardize rates according to the number of assessments completed. Consistent with prior work,^7,26^ we adjusted our models for baseline QIDS score. We explored PSE provider effects by estimating regression models to determine variation in participant outcomes across providers. We verified our main outcomes using multiple imputation techniques for missing data.^11^ We conducted all analyses described with SAS statistical software version 9.3 (SAS Institute Inc).

To evaluate the association between receipt of PSE, potentially mediating factors, and depressive symptom elevations, we constructed initial path models with single mediating variables using Mplus software version 7.3.1 (Muthen & Muthen). The primary purpose of these single mediator models was to present unadjusted estimates to provide a window into the degree of confounding in our subsequent main multivariate models. Receipt of PSE was modeled as a binary indicator variable; mediating factors were modeled as continuous variables; and depression symptom elevation over time was modeled as a count variable with an offset for the number of completed outcome assessments. Thus, the first part of the path model (linking PSE receipt to mediators) represented a linear model, and the second part (linking mediators to depressive symptom elevation) represented a Poisson model.^41^ To allow for comparisons across mediators, we modeled all mediating variables as standardized change scores, so that each participant had 2 change scores: 1 reflecting the time segment between baseline and 6 months of follow-up and the other between 6 months and 12 months. Depressive symptom elevations that occurred only within concurrent time segments were regressed against standardized change scores in mediator variables. Because there were no differential associations across the 2 time segments, the segments were combined into a single overall model with multiple observations per participant.

These models allowed us to differentiate and test the statistical significance of the direct from indirect (mediated) effects of PSE on rates of depressive symptom elevations. All path models were adjusted for baseline depression score and Head Start site; our fully adjusted models, constructed using the techniques described by Muthen and colleagues,^42^ also accounted for covariance among mediators (thus treating each of the mediating variables as potential confounders for one another). From a fully adjusted multivariate model that contained all theory-based, potentially mediating factors, we retained for further analysis in a more parsimonious model only those mediators that were associated with either receipt of PSE or depressive symptom elevations at the *P* ≤ .10 level. All *P* values were 2-sided.

Because there was no evidence of variation in outcome by PSE provider, and because imputing missing data did not change our results, we conducted all mediation analyses without adjusting for provider and we did not impute missing data for this purpose.

We estimated our sample size to provide power to test a clinically significant difference across intervention groups on the composite problem-solving measure and rate of symptom elevations. We did not power the study to detect signal in factors that mediate PSE’s impact.

## Results

### Enrollment

**Figure 1.**
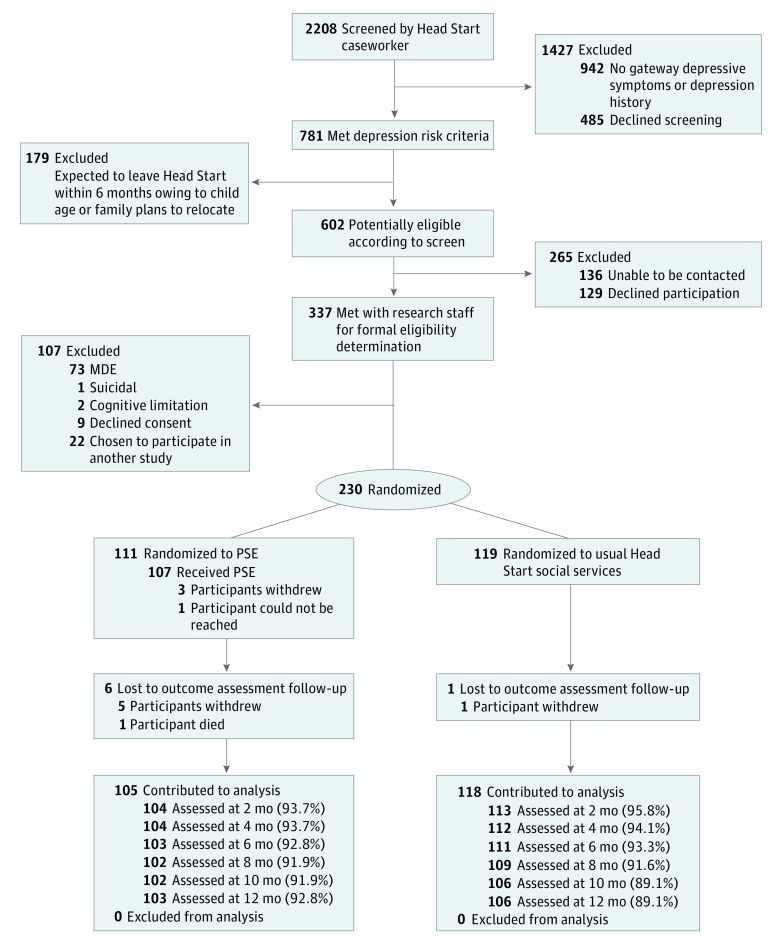
CONSORT Diagram From Silverstein et al.^11^ MDE indicates major depressive episode; PSE, problem-solving education.

We screened 2208 mothers; 602 potentially eligible mothers met depression risk criteria. Of these, 136 could not be contacted and 129 refused participation. Research staff met with 337 mothers for final eligibility determination, of whom 98 were ineligible (met criteria for MDE, were suicidal, had cognitive limitations, or were participating in another study); 9 declined informed consent. We enrolled 230 mothers (Figure 1).

### Baseline Characteristics

Hispanic mothers represented the majority of our sample (152 of 230 [66.1%]). The mean (SD) age of participants was 31.4 (7.3) years. Baseline mean (SD) depressive symptom scores were balanced between groups: 8.11 (5.20) in the PSE group vs 7.59 (4.38) in the usual service group. Baseline scores in all potentially mediating variables appeared to be balanced across intervention groups without any clinically meaningful differences (Table 1).

**Table 1.** Baseline Characteristics of Participants

Characteristics	Problem-Solving Education (n = 111)	Usual Head Start Services (n = 119)
Demographic characteristics		
Age, mean (SD), y	31.42 (7.08)	31.30 (7.53)
No. of children, mean (SD)	2.45 (1.29)	2.14 (1.21)
Race, No. (%)		
Black	37 (33)	44 (37)
Asian	0 (0)	3 (3)
White	28 (25)	33 (28)
Other, including multiracial	46 (41)	39 (33)
Hispanic, No. (%)	75 (68)	77 (65)
Education, No. (%)^a^		
Less than high school (including GED)	57 (52)	39 (33)
High school diploma	16 (14)	47 (40)
Some college	28 (25)	25 (21)
College degree or higher	9 (8)	8 (7)
Single-parent household, No. (%)	67 (60)	69 (58)
Potential intervention mediators, mean (SD)		
Social Problem Solving Inventory score^b^	13.04 (2.83)	13.02 (2.63)
Pearlin Mastery Scale score^c^	7.92 (2.21)	7.82 (2.09)
Rosenberg Self-esteem Scale score^d^	20.77 (5.13)	20.66 (4.95)
Behavioral Activation for Depression Scale score^e^	99.95 (24.98)	99.53 (21.93)
Avoidant coping score^f^	0.69 (0.64)	0.62 (0.66)
Problem-focused coping score^g^	1.65 (0.74)	1.76 (0.77)
Social coping score^h^	1.24 (0.89)	1.40 (0.88)
Perceived Stress Scale score^i^	2.86 (0.55)	2.79 (0.55)
Mental health measures		
QIDS score, mean (SD)^j^	8.11 (5.20)	7.59 (4.38)
QIDS score ≥11, indicating moderately severe depressive symptoms, No. (%)	36 (32)	34 (29)
Beck Anxiety Inventory score, mean (SD)^k^	12.08 (10.61)	12.07 (10.25)

Abbreviations: GED, general equivalency diploma; QIDS, Quick Inventory of Depressive Symptoms.

^a^ Data were missing for 1 participant in the problem-solving education group.

^b^ Scores range from 5.8 to 19.0, with higher scores indicating better problem-solving ability.

^c^ Scores range from 4 to 14, with higher scores indicating better mastery.

^d^ Scores range from 7 to 30, with higher scores indicating better self-esteem.

^e^ Scores range from 20 to 144, with higher scores indicating greater behavioral activation in the context of depression.

^f^ Measured using the Brief COPE. Scores range from 0 to 2.8, with higher scores indicating greater tendency to cope by avoiding problems.

^g^ Measured using the Brief COPE. Scores range from 0 to 3, with higher scores indicating greater tendency to cope by focusing on problems.

^h^ Measured using the Brief COPE. Scores range from 0 to 3, with higher scores indicating greater tendency to cope by engaging a social network.

^i^ Scores range from 1.1 to 4.6, with higher scores indicating greater level of perceived stress.

^j^ Scores range from 0 to 20, with higher scores indicating a greater level of depressive symptoms

^k^ Scores range from 0 to 46, with higher scores indicating a greater level of anxiety symptoms.

### Intervention Delivery and Fidelity

There were 111 mothers in the PSE group. Of a possible 6 PSE sessions, the mean (SD) number completed was 4.64 (2.06). Of 54 audiotaped PSE sessions, 28 met criteria for good model fidelity (≥80% of PSE components delivered) and 25 met criteria for excellent fidelity (≥90%). One audio file was damaged.

### Depressive Symptoms

Scores on the QIDS were missing from 102 of 1380 possible follow-up assessments (7.4%). In the full sample, the mean (SD) number of moderately severe symptom elevations in the PSE group was 0.84 (1.39), compared with 1.12 (1.47) in the usual service group, for mean (SD) rates, considering a possible 6 outcome assessments, of 0.17 (0.28) and 0.28 (0.35), respectively. This difference produced an adjusted rate ratio (aRR) of 0.60 (95% CI, 0.41-0.90) in favor of PSE. There was no evidence of variation in outcomes by PSE provider. Imputing missing data did not change our results. These outcomes have been reported previously.^11^

### Analyses of Single Mediating Variables

In a series of initial path models in which all potential mediators were tested individually, perceived stress and behavioral activation were associated with both PSE participation and depressive symptom elevation (Table 2 and eTable in Supplement 2 [unstandardized values]). On average, PSE participants had perceived stress change scores that were 11% of a standard deviation greater than controls (95% CI, −0.19 to −0.03), and 1 SD of improvement in perceived stress generated an aRR of 0.38 (95% CI, 0.30-0.47) for depressive symptom elevations. Similarly, PSE participants had behavioral activation change scores that were 15% of a standard deviation greater than controls (95% CI, 0.01-0.30), and 1 SD of improvement in behavioral activation generated an aRR of 0.74 (95% CI, 0.65-0.83) for depressive symptom elevations.

**Table 2.** Single Mediator Pathways

Mediator	Path Coefficient for Change Score Associated With Participation in PSE (95% CI)	Standardized Path Coefficient for Change Score Associated With Participation in PSE (95% CI)^a^	Rate Ratio of Depression Elevations per Standard Deviation Difference of Change in Mediator Score (95% CI)
Overall problem-solving ability	0.17 (−0.13 to 0.47)	0.08 (−0.07 to 0.23)	0.80 (0.71-0.90)
Mastery	−0.13 (−0.45 to 0.19)	−0.05 (−0.19 to 0.09)	1.14 (1.01-1.29)
Self-esteem	0.46 (−0.13 to 1.05)	0.11 (−0.03 to 0.25)	0.79 (0.69-0.91)
Behavioral activation	3.10 (0.11-6.09)	0.15 (0.01-0.30)	0.74 (0.65-0.83)
Avoidant coping^b^	−0.02 (−0.12 to 0.08)	−0.02 (−0.17 to 0.13)	1.14 (1.01-1.30)
Problem-focused coping	0.12 (0.02-0.22)	0.17 (0.03-0.31)	1.04 (0.92-1.17)
Social coping	0.10 (−0.03 to 0.23)	0.12 (−0.03 to 0.27)	1.02 (0.90-1.15)
Perceived stress^b^	−1.45 (−2.53 to −0.37)	−0.11 (−0.19 to −0.03)	0.38 (0.30-0.47)

Abbreviation: PSE, problem-solving education.

^a^ Coefficients represent percentage of a standard deviation in change of a mediator score.

^b^ Negative values for change indicate improvement.

The only other mediator associated with PSE participation was problem-focused coping. All mediators, except for problem-focused and social coping, appeared to be associated with the rate of depressive symptom elevations when covariance among them was not accounted for.

### Mediation Analyses Accounting for Covariance Among Mediators

**Figure 2.**
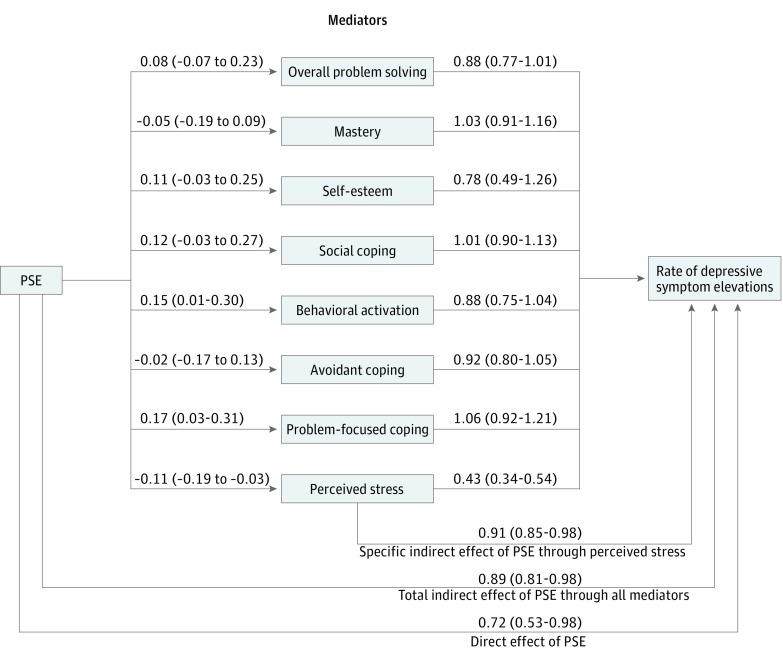
Full Multivariate Model Coefficients linking problem-solving education (PSE) to mediators convey the adjusted difference in percentage of a standard deviation of change between those receiving PSE and those not receiving PSE. The values shown in parentheses are 95% confidence intervals. For perceived stress and avoidant coping, a negative value conveys a more positive outcome; for all other mediators, a positive value conveys a more positive outcome. Estimates shown linking mediators to depressive symptom elevations convey the adjusted rate of depressive symptom elevations associated with an incremental 1 SD change mediator score.

In the full multivariate model (Figure 2 and eFigure 1 in Supplement 2 [unstandardized values]), only perceived stress was associated with both PSE participation and incidence of depression symptom elevation. On average, PSE participants had adjusted perceived stress change scores that were 11% of a standard deviation lower than controls (95% CI, −0.19 to −0.03), and 1 SD of improvement in perceived stress generated an aRR of 0.43 (95% CI, 0.34-0.54) relative to depression symptom elevations. These point estimates did not differ appreciably from the single mediator models, suggesting that the relationship between perceived stress and depression symptom elevation was not confounded by other mediators.

In the full multivariate model, PSE participants had adjusted behavioral activation change scores that were, on average, 15% of a standard deviation greater than controls (95% CI, 0.01-0.30); however, improvement in behavioral activation was not associated with a lower rate of depressive symptom elevations (aRR, 0.88; 95% CI, 0.75-1.04). Similarly, PSE participants had adjusted problem-focused coping change scores that were 17% of a standard deviation greater than controls (95% CI, 0.03-0.31); however, improvement in problem-focused coping was not associated with a lower rate of depressive symptom elevations (aRR, 1.06; 95% CI, 0.92-1.21). Although a better score on the Social Problem Solving Inventory (overall problem solving) appeared borderline protective against depression symptom elevations (aRR, 0.88; 95% CI, 0.77-1.01), PSE did not alter problem solving inventory scores (8% of a standard deviation difference across intervention groups; 95% CI, −0.07 to 0.23). Mastery, self-efficacy, avoidant coping, and social coping were associated with neither PSE participation nor rate of depression symptom elevation.

**Figure 3.**
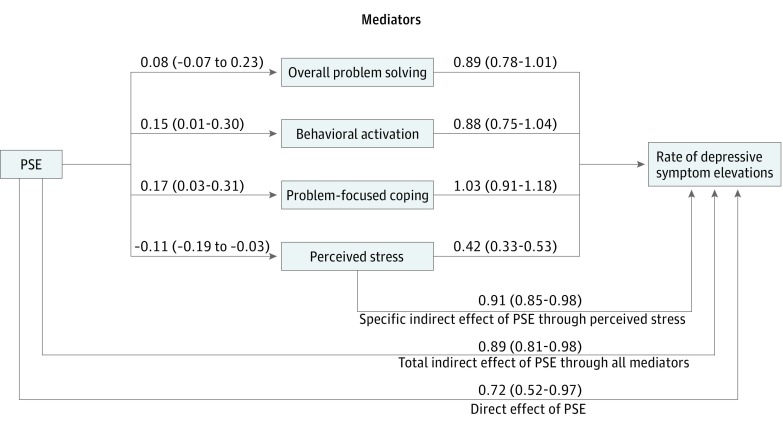
Parsimonious Multivariate Path Model Coefficients linking problem-solving education (PSE) to mediators convey the adjusted difference in percentage of a standard deviation of change between those receiving PSE and those not receiving PSE. The values shown in parentheses are 95% confidence intervals. For perceived stress, a negative value conveys a more positive outcome; for all other mediators, a positive value conveys a more positive outcome. Estimates shown linking mediators to depressive symptom elevations convey the adjusted rate of depressive symptom elevations associated with an incremental 1 SD change mediator score.

In a more parsimonious path model (Figure 3 and eFigure 2 in Supplement 2 [unstandardized values]) including only overall problem-solving scores, behavioral activation, problem-focused coping, and perceived stress, individual regression coefficients did not change substantially from the full, theory-driven model. In the parsimonious model, only perceived stress was associated with both PSE participation and depression symptom elevation (aRR, 0.42; 95% CI, 0.33-0.53). In this model, PSE generated a total aRR of 0.64 (95% CI, 0.45-0.89) for depressive symptom elevations (consistent with our previously reported results).^11^ The direct effect of PSE on depressive symptom elevation (aRR, 0.72; 95% CI, 0.52-0.97) was greater than its indirect effect explained by either the full constellation of mediators (aRR, 0.89; 95% CI, 0.81-0.98) or by improvement in perceived stress alone (aRR, 0.91; 95% CI, 0.85-0.98).

## Discussion

A growing body of evidence has demonstrated that depression is a preventable illness.^43^ Among women with young children, successful prevention approaches have primarily involved versions of interpersonal or cognitive-behavioral therapy.^44,45,46,47^ Problem-solving interventions, which are based in part on a cognitive-behavioral model, have a growing evidence base.^7,8,9^ Consistent with other depression interventions that have included problem-solving components,^7,12,13,48^ recent work has demonstrated that PSE substantially reduces the rate of symptomatic person-time over a full year.^11^ According to the present study, most of this impact appears to have been a direct effect of PSE on depressive symptoms. However, it also appears that a substantial part of PSE’s effect is mediated through a constellation of other factors, most notably the reduction of perceived stress. Although PSE appears to enhance behavioral activation and problem-focused coping, these intervention targets do not appear to be associated with symptom improvement.

This constellation of findings is consistent with PSE’s underlying theoretical model, the relational problem-solving model of stress, which posits that improving, or buffering an individual from, daily life stress can improve depressive symptoms.^10^ Gaining understanding into how behavioral interventions work is important for at least 2 reasons: first, understanding the psychological and behavioral targets that such interventions engage can lead to model refinements that maximize effect; and second, understanding mechanisms of action will ultimately allow for the differentiation of core intervention components from adaptable ones, a process critical for intervention implementation. Our specific findings are consistent with prior studies of problem-solving interventions that have improved depressive symptoms in the absence of measurable differences in problem-solving skills.^7,39^ However, whereas prior work has suggested that problem-solving models work by breaking down avoidant coping patterns,^16^ we found no signal that PSE engaged that particular intervention target.

The finding that PSE’s direct effect on depressive symptoms appears greater than its mediated effect can be interpreted in 2 ways. The first is that our measurement strategy could have underestimated the role of stress as a mediating factor. In this case, the Perceived Stress Scale may have incompletely captured the domains of stress we sought to measure. If we had measured a more specific dimension of stress by honing in on stress related to parenting or depression, then it is possible we could have detected a more powerful mediating effect. This argument can be extended to other mediating constructs such as problem-solving ability and behavioral activation, which are difficult to measure with concise, time-efficient scales. The second interpretation is that we have yet to fully understand problem solving’s primary mechanism of action; in this case, some of PSE’s purported direct effect may actually be an unmeasured mediated effect.

### Limitations

Our study has a number of limitations. First, a mediation analysis by path modeling is not an experimental design. The way to definitively test alternative mechanistic pathways is to compare the effectiveness of different (and often simplified) versions of an intervention; this was beyond the scope of our clinical trial. Second, we did not power the study specifically to detect signal in factors that mediate PSE’s impact; thus, it is possible that our findings concerning behavioral activation and problem-focused coping (which, though statistically significant, are on the border of conventionally accepted levels of statistical significance) may be spurious findings, and that undetected mediation pathways exist even among the variables we tested. Third, although our mediation analysis supports the paradigm that buffering an individual from stress can lessen depressive symptoms, it still leaves unanswered exactly how that buffering occurs in the context of PSE. Our mediation models are specific to the impact of problem solving on depressive symptoms and do not necessarily relate to other, potentially important outcomes related to behavior change, for which behavioral activation or problem-focused coping skills may play a more substantial mediating role.

## Conclusions

Our study contributes to the literature on problem solving’s role as a pragmatic depression prevention strategy and potentially advances it along the trajectory from efficacy testing to effectiveness testing and implementation. Insight into PSE’s mechanism of action, specifically in the context of an efficacy trial, can plausibly lead to intervention refinements for both PSE proper and other related intervention models. In turn, such refinements can help develop simplified intervention strategies that are more likely to stand up to real-world effectiveness testing, and ultimately to dissemination and implementation.

## Notes

Supplement 1. **Trial Protocol**

Supplement 2. **eTable.** Single Mediatory Pathways With Unstandardized Values **eFigure 1.** Full Multivariate Model With Unstandardized Values **eFigure 2.** Parsimonious Multivariate Path Model With Unstandardized Values
