# Supplementary material for: Problem-Solving Education to Prevent Depression Among Low-Income Mothers: A Path Mediation Analysis in a Randomized Clinical Trial
Source: JAMA Netw Open. 2018 Jun 29;1(2):e180334. doi: 10.1001/jamanetworkopen.2018.0334 (PMC6324419; doi:10.1001/jamanetworkopen.2018.0334)
Supplement: Supplement 1. — Trial Protocol [file jamanetwopen-1-e180334-s001.pdf]

STUDY PROTOCOL – PREVENTING MATERNAL DEPRESSION IN HEAD START

General Information
**Full title:** Preventing Maternal Depression in Head Start
**Boston University Medical Center Departments involved in study:** Pediatrics, Community Health Sciences, Biostatistics
**Principal Investigator**: Michael Silverstein, MD MPH
**Co-Investigators**: Mark Hegel, Emily Feinberg, Judith Bernstein, William Beardslee
**Research staff personnel**: Clarissa Andre, Diane Garzon Arbelaez, Julianna Brody-Fialkin, Lynn Cadena, Catherine Alice Cook, Shirley De La Cruz-Garcia, Yaminette Diaz-Linhart, Sharita Fauche, Ivys Fernandez-Pastrana, Catalina Obregon Folch, Adriana Giron, Andrea Medeiros, Michelle Claire Pellicer, Jenna Sandler, Meagan Wyllie
**Conflict of Interest Disclosures**: none
**Funding Source**: National Institute of Mental Health: R01MH091871
**Collaborating Institutions Engaged in Research:** Action for Boston Community Development, FWA 00016040 (IRB Authorization Agreement in Study Binder; ABCD Contract, Milagros Ramirez)
**Collaborating Institutions NOT Engaged in Research:** Children's Hospital, Boston; Dartmouth-Hitchcock Medical Center
**IRB of record:** Boston University Medical Center
**Last IRB Approval Date: 3/02/17**
**IRB Expiration: 3/15/18**
**Protocol last confirmed or amended: 1/27/18 (confirmed)**

© 2018 Silverstein M et al. JAMA Network Open.

Study Summary

This study is a community-based randomized trial of an intervention to prevent maternal depression in Head Start. The intervention is Problem Solving Education (PSE). We are conducting an efficacy RCT with a total enrollment goal of 230 Head Start mothers at risk for depression (not with major depressive episode, MDE).

This study is operationally linked to two other pilot studies, which draw their participants from the same recruitment infrastructure: a pilot study of how to engage a separate population of Head Start mothers *with MDE* into care; and an administrative supplement-funded pilot of an abridged, or simplified, version of PSE (referred to as PSE-lite), delivered to Head Start mothers with the same characteristics of those participating in the larger clinical trial (a schematic representation of how these three studies interact is provided in the study binder). In the current RCT, we use PSE to prevent depression in mothers at risk for depression.

Considering the three studies, the following represent key steps in the overall protocol:

1. Screening and risk stratification before the written informed consent process to determine if a subject, at baseline, is 'at risk' for depression, or has MDE.
2. Of the 'at risk' subjects, most will be offered participation in the prevention RCT. Of these, half will receive PSE. The other half will not receive PSE, but will be referred to further care when appropriate per usual Head Start mechanisms (i.e. the control group will receive usual Head Start services).
3. A small number of ‘at risk’ subject will be offered participation in the supplementary pilot of PSE-lite. These individuals represent participants over and above the 230 projected to enroll in the trial.
4. Those in MDE will be routed to Engagement with Care pilot study. This study aims to navigate depressed mothers into more definitive mental health care; it is a separate study, but will share research staff (for both recruitment/enrollment and outcome assessment.
5. If at any point during the prevention study, any mother has high depressive symptoms, we will offer a referral to more definitive mental health care.

Background/Rationale/Purpose

Depression is common among young, low-income mothers and has negative repercussions on children. Additionally, this population of mothers lacks access to quality mental health services – making a prevention intervention potentially important. We are conducting a multi-year RCT of a Head Start-based intervention model developed as part of Dr. Silverstein's and Dr. Feinberg’s K23 awards. PSE represents an integrated identification and intervention system based on the construct of screening, brief intervention, and referral to treatment (SBIRT). Within the case management services of Head Start, eligible mothers will be randomized to receive either usual care or PSE. The basic steps of PSE are: screening to determine the severity of one's depressive symptoms; a problem-solving intervention

© 2018 Silverstein M et al. JAMA Network Open.

for those at risk for depression; and referral to more definitive services for those initially in the at risk group, who have worsening symptoms over time.

The specifics of PSE have been modified and field-tested over the previous four years (primarily in Head Start, under another BUMC IRB protocol H-25084). Activated referrals to further care incorporate principles of motivational enhancement that have been demonstrated to improve engagement with care in other settings and for other medical conditions. We will conduct an efficacy RCT, involving 230 Head Start mothers. Over a 12-month follow up period, we aim to:

1. Improve mothers' problem solving skills (primary outcome)
2. Decrease the incident rate of episodes in which mothers experience moderate to severe depressive symptoms and reduce depressive symptoms over time (primary outcome)
3. Increase mothers' likelihood of engaging with primary care or specialty mental health services if referred (secondary outcome)
4. Improve parental functioning, as assessed by children's missed Head Start school days (secondary outcome).

Subjects

Inclusion Criteria

Based on the paradigm of the 1994 IOM report on preventing mental disorders, we have designed PSE to be a risk-prevention intervention - designed to prevent depression among mothers *known to be at risk*. The IOM calls this a 'selective' prevention strategy. PSE has already been adapted for mothers of young children, who represent our population of interest.

Summary of inclusion criteria

1. Family enrolled in Head Start, and child expected to attend Head Start activities for at least 6 months of the 12- month follow-up period.
2. Family without imminent plans to relocate
3. Mother is comfortable in English or Spanish.
4. Parent/guardian who has a child that attends Head Start
5. Child attending Head Start and is between the ages 0-5
6. Screen positive to 2-question depression screener (PHQ2), or have a past history of depression
7. Screen Negative for current MDE using the M.I.N.I MDE Module.
8. Ability to provide informed consent.
9. Not suicidal

Details of Inclusion Criteria

Based on previous work aiming to prevent major depression, we will initially select at-risk mothers

© 2018 Silverstein M et al. JAMA Network Open.

using two criteria: 1) a recent history of depression, or 2) current subsyndromal depressive symptomatology. This screening component (a 5 question screener) will be embedded within Head Start caseworkers' Strength and Needs Assessment of Head Start families. The primary purpose of this is to screen people *into* the study.

Mothers who are positive on this initial recruitment screen will be called by research staff and will be asked permission for a second screen to determine if they are currently (past 2 weeks) in a Major Depressive Episode using the M.I.N.I MDE Module. The primary purpose of this is to screen people *out of* the study.

Only mothers who are both positive on the 5 question recruitment screen AND who are MDE negative in the past 2 weeks will engage in formal written informed consent.

Immediately after the written informed consent process (described later), we will use the MacArthur Competence Assessment Tool for Clinical Research (MacCAT-CR) to determine the potential subject's capacity to consent to research. This screening tool will screen out any mothers who are psychotic, or impaired in their capacity to participate in a research study. The MacCAT-CR thus serves the dual purpose of confirming one's ability to provide informed consent and screening out thought-disordered individuals from a study that is not appropriate for them.

Next, we will use the MacAurthur Suicide Screening Questions to assess suicidal ideation at low, intermediate or high risk. We will only accept mothers into the study who are at low or intermediate risk for suicide. This means that either mothers with no current thoughts or with current thoughts but no plans of suicide will be eligible for participation. The reason for this exclusion criterion is participant safety. Our plans to manage a crisis brought on by the expression of suicidal ideation are also detailed in our attached protocols.

Exclusion Criteria

<u>Summary of Exclusion criteria</u>

1. High suicidal ideation (McArthur Suicide Screen)
2. Cognitive limitation (McCAT-CR)
3. Psychosis
4. No known risks for depression (based on recruitment screener)
5. Positive for MDE on the M.I.N.I. MDE Module (Note that MDE Positive individuals will be refered to participate in study outlined in protocol H-31676).
6. Mothers who have plans to leave the State of Massachusetts and/or the Country during the 12-month period of the study for more than 2 months will be excluded from the study for safety and research follow up reasons. These mothers will be included in the next wave of recruitment.

<u>Details of Exclusion Criteria</u>

Mothers who are NOT at risk for depression based on current symptoms and past history of depression illness be excluded from the study based on the recruitment screen. These mothers would not benefit

© 2018 Silverstein M et al. JAMA Network Open.

from a prevention trial (PSE) since they are not at risk for depression. Similarly, mothers with MDE will not be eligible.

In addition, we will exclude mothers who cannot give capacity to consent because of either cognitive limitation or psychosis by using the MacCAT-CR. We will also exclude mothers with high suicidal ideation by using the MacAurthur Suicide Screening Questions. Both of these measures have been used widely in other research studies to protect vulnerable populations.

Subjects – Demographic Characteristics and Vulnerabilities

For regulatory purposes, we will indicate to the IRB that we will be interacting with the following vulnerable populations and populations requiring special protections:

1. Adolescents, ages 15-17
2. Adults, ages 18-64
3. Children, ages less than 7
4. Minors independently making their own healthcare decisions
5. Pregnant women
6. Women of child bearing potential
7. Women with depression or symptoms of depression could be considered a vulnerable population

Children less than 7 years constitute study subjects because parents and Head Start teachers will be asked to fill out the Social Skills Rating System with reference to 3-5 year-old children of the adult study subjects. Additionally, some of the women recruited in our study may be of childbearing potential, may be pregnant or may be emancipated minors (e.g. teen mothers). The following will be explained in our IRB protocol:

INFORMED CONSENT: The consent form will be explained in very basic language, and mothers will be given ample opportunity to ask questions and/or opt out of the study. We will ask permission for teachers to answer questions about their children, and they may still be in the study and not give us permission to do so.

CONFIDENTIALITY: We will apply for a federal certificate of confidentiality. Mothers will also be informed that all data collected will be strictly confidential. All data collected will be stripped of identifiers and assigned a unique study code.

UNDUE COERCION: All subjects will be explained that their participation is completely voluntary, and that they have the right not to participate. They will be explained that even if they do not participate, project staff will do everything it can to help them with any symptoms of depression that they may have. They will be explained that should they not want to participate, no benefit that they have will be affected. They will be explained that they may drop out of the study at any point, should they chose to do so, and that no questions will be asked. Study personnel will also be happy to assist them in accessing further care.

PROTECTIONS FOR WOMEN OF CHILD BEARING POTENTIAL/PREGNANT WOMEN Women of child bearing potential or pregnant women will be entitled the same protections as listed above. We

© 2018 Silverstein M et al. JAMA Network Open.

foresee no extra risk of the study for a woman of child bearing potential than it is to anyone else.

Design

At present, all Head Start mothers are given social work and case management services and education by the Head Start team regarding the importance of physical and mental health care. Mothers are screened for mental health needs during their "strength and needs assessment" by the case management staff and given referrals to care when necessary. However, there is no systematic depression prevention strategy, and there is no tested, evidence-supported mechanism to get depressed parents quality mental health care.

Study design. Parallel group, RCT involving mothers whose children attend Head Start - a comprehensive preschool program for low-income children birth to 5. Eligible mothers will be randomized to receive PSE or standard of care at Head Start. Both groups will be followed for 12 months.

Participants. 230 Head Start mothers (115, PSE; 115, control) will be enrolled in the at-risk stratum.

Randomization. The unit of randomization will be the mother. Investigators and data collectors will be blinded to study allocation. We could have chosen a number of study designs, including cluster randomization by caseworker or Head Start center. Cluster randomization, however, has its own set of methodologic shortcomings, notably post-randomization selection bias. Because we also expect high intra-class correlation among families within the same Head Start center, a cluster design on this basis would create unrealistic sample size requirements. Conversely, the only substantial drawback of a parallel design would be a conservative bias in favor of the control arm due to intervention spillover. However, because control subjects will not have access to trained educators, we anticipate such effects to be minimal. Randomization will occur independently within each Head Start site, within strata defined by the presence or absence of depression history. We will randomize 1:1 within randomly varying blocks of 2 and 4.

Procedure

1. Boston's ABCD Head Start will obtain a Federalwide Assurance (FWA00016040), on which it will designate the BUMC IRB. All personnel at ABCD Head Start deemed to be engaged in research will take the online NCI course in human subjects protections, and certificates will be emailed to the BUMC IRB. Additionally, such personnel will receive project-specific training directly from Dr. Silverstein (PI) and Dr. Feinberg (co-I). We currently have an IRB approved IAA with ABCD Head Start.
2. The trial will be registered on clinical trials.gov prior to enrolling participants.
3. The PI and study coordinator will update NIMH’s RMR (Recruitment Milestone Report) per NIMH’s schedule.
4. Participants: 230 at-risk Head Start mothers will be enrolled according to the eligibility criteria indicated in previous section.

© 2018 Silverstein M et al. JAMA Network Open.

5. Procedures pre-written informed consent

   a. Verbal Consent 1 and Recruitment Screen: Verbal consent will be used prior to screening for eligibility with a temporary study code; Head Start caseworkers will conduct this initial round of screening and determine with study staff IRB-compliant modes of communication to make staff aware of mothers with positive screens. Only eligible subjects' names and telephone numbers will be recorded during screening, in addition to the screening results.

   b. Verbal Consent 2 and MDE Screen: Research assistants will set up appointments with eligible mothers (those positive in recruitment screen) to conduct a second verbal consent and screen to determine if eligible mother is currently in a Major Depressive Episode.

   c. Only eligible mothers (positive on recruitment screen and negative on MDE screen) will be offered written informed consent.

   d. For those individuals who refuse screening, are not eligible or opt not to participate, we will record only their temporary study code on the recruitment screen. We will submit this list of temporary study codes to Head Start personnel for two reasons: 1) to avoid inadvertent re-approaching; and 2) so that Head Start can supply the study team with an anonymized demographic description of this population with a de-identified list of demographic characteristics, (Attached in the study binder). Specific variables will include only those that Head Start keeps for programmatic reasons (the equivalent of existing clinical data): age, language spoken, place of birth, race, ethnicity, education, work status, marital status, household type (single or double-headed household), number of people in household, living situation, income, health insurance status, type of government assistance.

4. Written Informed Consent: After the two screens are administered and the potential subject is found to be eligible, the subject will be enrolled (if willing) into either the RCT or into a small supplemental pilot study of 'PSE Lite.' Immediately following the signing of the consent form, the following will happen:
   a. Subjects will be administered either the MacCAT-CR immediately after the consent form in order to ensure that subjects with limited cognitive capacities and/or major mental illness, including psychosis, are safeguarded from being in the study.
   b. Subjects will be screened for suicidal ideation in order to safeguard subjects with high suicidal ideation. Our crisis protocols will be activated as necessary (Crisis Management Plan, Domestic Violence Protocol, Referral of Clients with Trauma or Substance Use – each protocol is provided in the study binder).

5. Once participants have completed the safeguards outlined in above, we will collect the following data from all mothers enrolled in the study *prior to* randomization. The interviews will be completed in private rooms at the Head Start center, or in subjects' homes (subjects' choice). The Baseline Survey will comprise the following valid and reliable scales:

   a. Children with Special Health Care Needs Screener

© 2018 Silverstein M et al. JAMA Network Open.

b. Perceived Stress Scale
c. M.I.N.I. Depression Module (current or recurrent Major Depressive Episode (We will use the data from the second verbal screen for this. We have added this permission to the written consent form.)
d. Quick Inventory of Depressive Symptoms. Beck Anxiety Inventory
e. Mental Health Service Use questions (adapted from Collaborative Psychiatric Epidemiology Surveys)
f. Medical Outcomes Study – Social Support Survey
g. Social functioning (Social Adjustment Scale-Self Report)
h. Problem Solving Questions (Social Problem Solving Inventory)
i. Substance Use (CAGE-AID and AUDIT)
j. Coping Questions (Brief COPE)
k. Rosenberg Self-Esteem Scale
l. Behavioral Activation for Depression Scale
m. Pearlin Mastery Scale
n. Stressful Life Events Questionnaire
o. Modified PTSD Symptom Scale
p. Demographics
q. Behavioral Activation Score

6. If randomized to the PSE group, we will employ a team of 4 to 6 research staff (at any given time) to serve as interventionists (referred to as educators or providers). The educators will deliver up to 6 PSE sessions with their clients, each session expected to take 30-45 minutes. Sessions will be delivered either weekly or biweekly, according to the wishes of the subject – with a full course of PSE expected to last 8 weeks. Sessions will be delivered at a location of the subject's choosing. In addition to PSE, intervention subjects will be periodically screened with the PHQ-9 as part of 3 out of the 6 PSE sessions. A score of 15 or greater will require the educator to refer the mother to more definitive mental health care. These referrals will be "activated," meaning that the PSE educators will employ motivational interviewing techniques to help the mothers follow through with the referrals. Lastly, educators will phone their clients monthly to conduct brief "booster session" check-in's.

   Request to audiotape a randomly selected PSE will be embedded in the initial consent form; this will be offered to all subjects for the sake of monitoring treatment fidelity. It will be made clear that subjects can refuse audiotaping but still partake in the rest of the study.

7. Control group: We have chosen our control group to best approximate "usual care" in Head Start. Thus, families will be followed by their Head Start caseworkers, and screened periodically for depression when deemed necessary by Head Start staff, per their usual routine. Referrals will be made

© 2018 Silverstein M et al. JAMA Network Open.

according to criteria specified in the grant application.

8. Outcome assessments: Each subject will have a 12-month follow up period, during which blinded research assistants will assess outcome measures. Follow-up data collection will begin 2 months after baseline because a full, 6-session course of PSE typically lasts 6-8 weeks. To limit attrition, we will employ a combination of monetary incentives and postcard reminders. Through these strategies, approved by the BUMC IRB in our pilot work, we have been able to maintain follow up rates > 85% across all relevant ongoing studies (there is a separate section on participant compensation in the protocol manual).

Baseline, 6-month, and 12-month assessments will take place in person with a staff RA. We will administer the QIDS and mental health service use questions on a bimonthly basis throughout the study period, and this will be done by telephone by a staff RA.

<u>The 6-month interview will comprise the following valid and reliable scales:</u>

a. Perceived Stress Scale
b. M.I.N.I. Depression Module
c. QIDS
d. Mental Health Service Use questions (adapted from Collaborative Psychiatric Epidemiology Surveys)
e. Social functioning (Social Adjustment Scale-Self Report)
f. Parenting Stress Index
g. Problem Solving Questions (Social Problem Solving Inventory)
h. Coping Questions (Brief COPE)
i. Pearlin Mastery Scale
j. Behavioral Activation for Depression Scale
k. RosenbergSelf-Esteem Scale

<u>The 12-month interview will comprise the following valid and reliable scales:</u>

a. Perceived Stress Scale
b. M.I.N.I. Depression Module
c. QIDS
d. Mental Health Service Use questions (adapted from Collaborative Psychiatric Epidemiology Surveys)
e. Social functioning (Social Adjustment Scale-Self Report)
f. Problem Solving Questions (Social Problem Solving Inventory)
g. Coping Questions (Brief COPE)
h. Pearlin Mastery Scale
i. Behavioral Activation for Depression Scale
j. Rosenberg Self-Esteem Scale
k. Recent Life Changes Questionnaire

© 2018 Silverstein M et al. JAMA Network Open.

l. Mood, anxiety and alcohol and other substance use, modules of the SCID

RA's will be trained not to leave telephone messages, and to introduce themselves as from "BMC" so as to ensure confidentiality.

9. At 6-months post randomization, parents and Head Start teachers will be asked to fill out the Social Skills Rating System (SSRS) with reference to all 3-5 year-old Head Start children of enrolled parents.
10. We will assess rate of absenteeism from Head Start for children of mothers who are in our study.
11. Dr. Silverstein or Mrs. Diaz Linhart will meet weekly with all educators for a supervision meeting. During this meeting, all cases will be discussed. In addition to this, Bill Beardslee - a board certified psychiatrist from Children's Hospital Boston - will be available for back up and for the rare, but possible, mental health emergency.

Outcomes

The following is pasted into the protocol directly from our NIMH grant application:

Outcome measures (*denotes primary outcome measure)

Outcomes	Operationalization of variable	Timing of data collection after baseline
**i. Problem Solving Skills***	**Calculated problem solving index from SPSI-R**	**6, 12 months**
**ii. Incident rate of moderate to severe symptomatic episodes***	**QIDS score ≥ 11, corresponding to 'moderate' symptomatology**	**2, 4, 6, 8, 10, 12 months**
**iii. Depressive symptoms over time***	**Mean QIDS scores over time**	**2, 4, 6, 8, 10, 12 months**
iv. Social and parenting functioning	Mean SAS-SR and PSI scores	6, 12 months
v. Receipt of mental health services	Standardized questionnaire	monthly recall
vi. Head Start attendance	Head Start records, analyzed as monthly absence rates	monthly reports

Sample Size Justification

Based on empiric longitudinal data that we have collected among a cohort of Head Start mothers, we describe the size of detectable interventional effects with adequate statistical power for each primary outcome. All estimates assume 80% power and a two-sided alpha of 0.05.

i. Problem solving skills. The mean problem solving (SPSI-R) score among mothers in our observational study is 12.2, (SD 2.5); women with ≥ moderate depression symptoms have a mean score of 14.7 (SD 2.2) and those with ≤ mild symptoms have a mean score of 9.8 (SD 2.8; p>0.0001). Using a two-sample t-test, a sample size of 100 per group will be able to detect a very granular one-point difference in mean SPSI-R scores between study groups (a standardized effect size of 0.4).

© 2018 Silverstein M et al. JAMA Network Open.

ii. Incident rate of moderate to severe symptomatic episodes. Using poisson regression analysis, with 100 subjects in each study group and a poisson rate of 1.2 spikes per 5 follow-up assessments in the control group (according to our current data), we can detect a reduction in the rate of spikes to 0.8 in the treatment group – a 33% reduction in symptomatic episodes.

iii. Depressive symptoms over time. In our longitudinal data, we have observed the following mean QIDS scores for each follow-up month and assume these values for the control group in the planned study: 8.2; 7.6; 7.5; 6.9; 7.2 (common SD, 5.9). We hypothesize that the following mean QIDS scores in the treatment group would be achievable and clinically significant: 7.2; 6.6; 5.5; 4.4; 4.2, resulting in a mean difference of 3 points in QIDS scores across study groups (note: a 3-point difference in QIDS scores, in the majority of cases, would move a subject across a clinical cutoff corresponding to mild, moderate or severe symptoms). Using a two group repeated measures design analyzed by generalized estimating equations or mixed linear models, noting within-subject correlations of 0.7 for values separated in time by one month and 0.6 for those separated for two months or more in our longitudinal analysis), we can detect the above differences in the means between groups over time with 100 subjects per study group.

Therefore, with an analyzable sample of 100 subjects per study group, we will be able to detect with adequate statistical power differences in problem solving scores, incident rates of elevated QIDS scores, and mean QIDS scores over time that are of clear clinical relevance. Based on our current data, we estimate that 20% of mothers approached will refuse participation; 70% will meet eligibility criteria; and we will experience a 15% attrition rate over the course of the study. Therefore, to ensure that we assess outcomes in 200 study subjects, we will approach 410 subjects and randomize 230. This sample size will be readily available from our ABCD Head Start sites, which average 100 families per site.

Data Analysis

Our analysis plan conforms to CONSORT guidelines. We will record the number of families approached, screened, ineligible, and refusing participation. We will assess the success of randomization by comparing baseline characteristics between groups. We will record subject attrition and note all adverse events. To estimate intervention effect, we will conduct an intention-to-treat analysis to compare the difference between intervention and control groups for all primary and secondary outcome measures. The following primary outcomes will be analyzed accordingly:

i. Problem solving skills. We will employ two sample t-tests to compare mean problem solving scores between groups. If significant baseline differences exist across groups, we will employ linear regression analysis to adjust for these differences.

ii. Incident rate of moderate to severe symptomatic episodes. We will employ Poisson regression techniques to compare rates of elevations in QIDS scores to ≥11 (moderate symptomatology) over the 12 months of follow-up.

iii. Depressive symptoms over time. We will use generalized estimating equations or mixed linear models to examine group-by-time effects on mean QIDS scores over the 12 months of follow-up.

© 2018 Silverstein M et al. JAMA Network Open.

Using generalized mixed models for each of these outcomes (to which the above analytic methods can be extended), we will also investigate the impact of Head Start enrollment site as a clustering effect. As an exploratory analysis, we will also examine additional clustering of PSE clients by educator.

This analysis plan, taken from our grant application, will be reproduced in our IRB application.

Risks and Discomforts (as communicated to the IRB)

Subjects may feel uncomfortable being asked questions about their mood.

Subjects will be made aware that they are under no obligation to fill out the screening instruments and under no obligation to engage in PSE sessions, or to follow through with a referral to mental health care.

Subjects may feel uncomfortable engaging in PSE. Again, they will be made aware that participation is strictly voluntary and that if they do choose to participate, they are under no obligation to have their session audiotaped (which we ask permission for, in order to assess intervention fidelity).

Subjects may feel uncomfortable being referred to mental health care. They will be made aware that they are under no obligation to give us permission to see if they have accessed mental health care if referred.

It is unlikely that PSE will precipitate a mental health emergency, as this type of activity is mainly educational in nature and does not explicitly address depressive symptoms. Rather, it will be offered to mothers who display such symptoms or are at risk for depression. PSE allows for clients to discuss their daily practical life problems, such as getting their children to school on time, paying their bills, juggling multiple tasks, and leaves it up to the client to pick the problem that she will work through with the caseworker. However, in the event that a mental health emergency occurs, we will have a crisis management plan in place. The crisis management protocol is available in the study binder.

Potential risks for uncovering moderate to severe depression, substance use, domestic violence, or trauma symptomatology. All clients in both the PSE and non-PSE groups will be administered the MINI depression module, trauma symptom scale, DV questions, and substance use questions. In both study arms, part of the intervention will be to refer clients with moderate to severe depressive symptoms to mental health care.

We have developed a resource list to give to any subject that is interested in accessing further care. We also plan on using Boston-based BEST team for potential mental health emergencies. Although many measures will be taken to ensure confidentiality, there is the possible risk of a breach of confidentiality. If such a breach is determined to have happened, we will contact all subjects whom the breach affected immediately, explain to them exactly what happened and to our best approximation who had access to the data. We will also offer to work with the subjects in any way they deem appropriate to rectify the situation.

Data Safety Monitoring Plan

Throughout the course of the study, the data will be monitored every week by Dr Silverstein in consultation with Dr. Cabral, statistician. Investigators will meet weekly with educators and research assistants to discuss cases and debrief on experiences. Monitoring will pay particular attention to adverse

© 2018 Silverstein M et al. JAMA Network Open.

events or events that had the potential to become adverse. In addition, as described above¨ we will continually monitor for events of suicidal ideation and activate a crisis management plan as necessary. In such an event, we will always notify the IRB and analyze if our plan functioned as expected.

Betsy Groves, LICSW will be our data safety monitor. Ms. Groves is the director of BMC's Child Witness to Violence Program and is an experienced therapist and mental health clinician. We will furnish Ms. Groves a de-identified dataset every 50 subjects with information on who required acute intervention and why in response to what study process/questionnaire item it was required. Ms. Groves will provide feedback to the study team regarding ongoing safety.

Potential Benefits

Based on previous evidence, it is likely that subjects' depressive symptoms will improve as a result of this intervention.

Women in a Major Depressive Episode or with high depressive symptoms will get referred to mental health care.

Women who are in a domestic violence situation, have substance abuse or dependence problems will also be referred to more definitive care through established medical and community-based organizations (see attached protocols)

Depression is a substantial public health problem, and preventing it among high risk mothers has substantial societal benefits in the areas of both health and education.

Risk/Benefit Ratio

We believe that the potential benefits of this project outweigh the risks. Previous data around access to care for young women with depressive symptoms consistently show that only a small minority of these women receive the necessary services. And since maternal depression is known to have ill effects for the child as well, leaving this condition untreated have repercussions for both the mother and child. If successful, this project will allow for a scalable and sustainable intervention for among a high risk population.

Recruitment Procedures

<u>Recruitment Screen</u>

Head Start caseworkers will obtain verbal consent from mothers to screen them for depressive symptomatology with the PHQ-2 and to assess recent depression history (Screening instrument is attached in study binder). This overlaps with current Head Start practice regarding screening mothers for depression.

<u>Major Depressive Episode Screen</u>

Mothers meeting either of these eligibility criteria will be offered to meet with a research assistant, who

© 2018 Silverstein M et al. JAMA Network Open.

will conduct a second verbal consent to screen for current MDE using the MINI MDE module.

Once the MINI is performed, research staff will obtain written consent, administer the in-person baseline survey, and randomly assign study allocation only if mothers meet the inclusion and exclusion criteria as outlined above (The baseline instrument is provided in the study binder).

Two important factors require us to temporarily record names of the potential subjects screened with this instrument:

- Screening for depression is part of normal Head Start practice through their "strength and needs assessment;" this initial screener will be done by Head Start staff. Therefore, Head Start sites have the option of using the PHQ2 AS BOTH A STUDY SCREENING INSTRUMENT AND A CLINICAL TOOL USED BY HEAD START STAFF. In order that it fulfill the latter purpose, an identifier is necessary - not for study personnel, but rather for Head Start personnel.
- Because Head Start staff will be conducting these initial screens (not study personnel), it is necessary that they be reviewed by study staff to most accurately determine ultimate study eligibility. We thus plan that study personnel review the screening results with Head Start staff.

We will therefore write the verbal consent form to explicitly inform potential subjects that this screening form will be reviewed one time by study personnel and that the information on the screen will not be recorded for any study purpose aside from what is mentioned above.

Furthermore, to safeguard confidentiality, the 'name' field will be replaced by a temporary study code. Head Start staff will keep the cross-walk between temporary codes and actual names in a separate location. The screening form, code, and cross-walk will all be destroyed as soon as study eligibility is determined.

A script for the initial case worker approach is will be kept in the study binder and provided to the IRB. We will also provide Head Start sites with study pamphlets to help introduce the research study in Head Start. These pamphlets will also be kept in the study binder and supplied to the IRB.

Recruitment Materials will all be available in the study binder.

Data from Screening Procedures

We will retain all data from screening procedures.

The initial screening data will be kept. We will include a de-identified data HIPPA waiver to collect pre-written informed consent aggregate data for ineligible mothers approached for the recruitment screen. In order to do this, we will attach a de-identified data form, which will be filled out by Head Start case managers. The following data will be collected: current gateway depression symptoms (depressed mood and anhedonia) and past depression history, depression symptoms based on DSM IV that may lead to a diagnosis of MDE.

Additionally, after written informed consent, we will retain data on ability to consent using Mac-CAT CR and level of suicidality based on MacArthur Suicide Screen.

Consent Procedures

RAs working for Dr. Silverstein will obtain consent and describe the informed consent process prior to definitively determining eligibility. Potential subjects will have as long as they wish to consider participating.<br><br>Our detailed consent and eligibility determination processes are as follows:<br><br>1. Head Start caseworkers will obtain verbal consent to screen clients for eligibility, as detailed above. We do this so mothers who are NOT at risk for depression-and therefore not appropriate for the study-will not be unnecessarily inconvenienced. This verbal consent and screen will occur in person or on the telephone. The latter mechanism is necessary because the mothers are frequently in phone contact with their Head Start caseworkers, but may not see them in person more than every month; and although the mothers will often come to Head Start to pick up their children, they usually do not see their caseworkers during this time (the verbal consent form is provided in the study binder). This process will be conducted by Head Start caseworkers.<br><br>2. Once caseworkers refer mothers who have screened positive for symptoms and/or a history, staff RA's will conduct a second verbal consent and screen additionally to see if the mother is currently in a Major Depressive Episode.<br><br>3. If mothers are eligible on recruitment screen AND are not currently in a Major Depressive Episode, written informed consent will be obtained by staff RAs. This will occur in person. The RA script for informed consent is provided in the study binder and will be supplied to the IRB. Research assistants will be trained to explain that written informed consent is required prior to eligibility screening due to the type of information collected. This is particularly important because if potential participants screen positive for suicidality, the study's crisis management protocol will be activated (available in binder and provided to the IRB).<br><br>4. Mothers will be explained during consent procedures that they will be randomized into either the supplement (H-31999) or the prevention trial (this protocol). If they are randomized into the prevention trial (this protocol), they will be explained that they will randomized a second time into the intervention or control group.<br><br>a. Because this protocol and the supplement to this protocol (H-31999) share the same consent form and we randomize only AFTER the consent form is signed, we will be using the consent form for THIS PROTOCOL (H-29448) for ALL participants, even those that will be later randomized to the supplement (H-31999). This is to maintain consistency between all consent procedures.<br><br>b. Any changes to the consent forms or procedures for this protocol or the supplement to this protocol (H- 31999) will be completed at the same time and no consent procedures will happen until both protocols are IRB aproved.<br><br>5. If we encounter an immature 15 or 16-year-old who would otherwise be potentially eligible, we will only take their informed consent if the person can actually give informed consent.<br><br>6. For all participants, we will use the MacArthur Competence Assessment Tool for Clinical research (MacCAT-CR) to determine participants' capacity to give informed consent. The MacCAT-CR instrument has been used widely in other clinical research studies and assesses a participant's

© 2018 Silverstein M et al. JAMA Network Open.

competence using four sections: understanding, appreciation, reasoning and expressing a choice. Please refer to study attachments for the customized MacCAT-CR.

For those who do not want to be screened and for those who ultimately opt not to participate, we will record only their temporary study ID, so that we do not inadvertently approach them again. We will submit this list of names to Head Start personnel, so they also know who has declined participation in the study and can give us de-identified demographic data as outlined in HIPPA section.

Waiver of Documentation of Informed Consent

Our waiver of documentation of informed consent applies only to the recruitment screening process and the Major Depressive Episode screening, and not any subsequent study procedures – for which written informed consent will be obtained.

Payment

All participants will receive a $100.00 gift card to Target after completion of the three in-person research interviews: 1) Baseline, 2) 6-month interview and 3) 12-month interviews. Participants who cannot complete in person 6 and 12 month interviews will be given the option of doing the interviews over the phone and compensated with a $50 gift card instead.

Participants will also receive $25.00 gift cards for completion of 15 minute over-the-phone research interviews: months 2, 4, 8 and 10.

The gift cards for the phone research interviews will be disbursed to participants at their next in-person interview in order to facilitate distribution and to limit lost or stolen gift cards. Mothers who choose to have 6 or 12 month interviews over the phone rather than in-person, will be delivered gift cards via their Head Start case manager or via registered mail.

In addition to payments for time compensation, participants without their own form of secure telephone communication will be offered a cell phone with a monthly plan. This will be given only to participants who need the cell phone as defined in our cell phone incentive plan. The primary purpose of this cell phone incentive is to develop a system to ensure quality follow up data and to ensure participant safety.

Attachments

All individual safety protocols, scripts, data collection instruments, and pamphlets will be available in the study binder and supplied to the IRB

Problem Solving Education Manual and Workbook

The PSE manual and workbook is available in the study binder; workbooks are available in English and Spanish.

© 2018 Silverstein M et al. JAMA Network Open.

Appendix – January 2018

The following appendix summarizes the content of an official letter written by the study PI to the National Institute of Mental Health in April 2010, specifying mediation and moderation analyses that were not included in the original grant application, in response to the NIH study section's summary statement (March 2010). This letter was requested by Program Official, Lauren Hill, PhD. The following summarizes the content of that letter. The letter itself is located in the study binder.

We outline an augmented analytic strategy to assess factors that have sound theoretical bases to either moderate or mediate the effect of PSE.

a. *Theory based moderators*: Individual coping styles can substantially modify the effect of a problem solving intervention,[1] as can the degree of baseline depressive symptoms.[2] We proposed evaluate moderation by these factors by performing stratified analyses, followed by formal testing of interaction terms in our statistical models.

b. *Theory based mediators*: According to our theoretical model (the Relational Problem Solving Model of Stress), improved problem solving skills and decreased perceptions of stress constitute likely intervention mediators; however, studies of other problem solving interventions have demonstrated impact in the absence of improved problem solving skills, problem resolution, or increased sense of mastery and self-control.[3] Given PSE's emphasis on goal setting and action planning, a plausible alternative mechanism is behavioral activation, which we proposed to assess through the Behavioral Activation for Depression scale.[4] We proposed to examine meditational effects using two potential techniques: the approach of Baron and Kenny[5] and the use of path analysis models.

We detailed specific measures to reflect these constructs; each of these measures is listed in the main body of the trial protocol.

Construct	Measure	Hypotheses
Behavioral Activation	The Behavioral Activation for Depression Scale (BADS) addresses four dimensions: activation, avoidance/rumination, work/school impairment, and social impairment; this measure thus spans the affective and functional domains.	Behavioral activation will partially mediate the effect of the intervention, particularly with regard to functional outcomes.
Mastery / Self-esteem	The Pearlin Mastery Scale, which measures the degree to which individuals perceive themselves as in control of their lives,[6] will be used in conjunction with the Rosenberg Self-esteem Scale.[7]	Self-perceived mastery will correlate with depression symptoms and problem solving orientation. Mastery will be impacted by intervention exposure but will not mediate its effect.
Cognitive components of problem solving	The COPE characterizes an individual's coping styles (e.g. denial, disengagement, planning.<br>The Social Problem Solving Inventory -Revised (SPSI-R) measures problem orientation and problem-solving skills in 5 dimensions: positive and negative orientation; avoidance;	Baseline coping style will modify the effect of the intervention: those with avoidant styles will benefit to a lesser degree than those with planning or reframing styles.<br>Problem solving skills will correlate with depressed mood and will partially mediate

© 2018 Silverstein M et al. JAMA Network Open.

	impulsivity; and rationality and thus primarily addresses the cognitive component of problem solving skills.	intervention effects.

References

1. Oxman TE, Hegel MT, Hull JG, Dietrich AJ. Problem-solving treatment and coping styles in primary care for minor depression. *J Consult Clin Psychol.* 2008;76(6):933-943.
2. Garber J, Clarke GN, Weersing VR, et al. Prevention of depression in at-risk adolescents: a randomized controlled trial. *JAMA : the journal of the American Medical Association.* 2009;301(21):2215-2224.
3. Mynors-Wallis L. Does problem-solving treatment work through resolving problems? *Psychol Med.* 2002;32(7):1315-1319.
4. Kanter JW, Mulick PS, Busch AM, Kristoffer SB, Martell CR. The Behavioral Activation for Depression Scale (BADS): Psychometric Properties and Factor Structure. *Journal of Psychopathology and Behavioral Assessment.* 2006;29(3):191-202.
5. Baron RM, Kenny DA. The moderator-mediator variable distinction in social psychological research: Conceptual, strategic and statistical considerations. *Journal of Personality and Social Psychology.* 1986;51:1173-1182.
6. Pearlin LI, Schooler C. The structure of coping. *J Health Soc Behav.* 1978;19(1):2-21.
7. Sinclair SJ, Blais MA, Gansler DA, Sandberg E, Bistis K, LoCicero A. Psychometric properties of the Rosenberg Self-Esteem Scale: overall and across demographic groups living within the United States. *Eval Health Prof.*33(1):56-80.

© 2018 Silverstein M et al. JAMA Network Open.
