# Supplementary material for: Problem-Solving Education to Prevent Depression Among Low-Income Mothers: A Path Mediation Analysis in a Randomized Clinical Trial
Source: JAMA Netw Open. 2018 Jun 29;1(2):e180334. doi: 10.1001/jamanetworkopen.2018.0334 (PMC6324419; doi:10.1001/jamanetworkopen.2018.0334)
Supplement: Supplement 2. — eTable. Single Mediatory Pathways With Unstandardized Values eFigure 1. Full Multivariate Model With Unstandardized Values eFigure 2. Parsimonious Multivariate Path Model With Unstandardized Values [file jamanetwopen-1-e180334-s002.pdf]

Supplementary Online Content

Silverstein M, Cabral H, Hegel M, et al. Problem-solving education to prevent depression among low-income mothers: a path mediation analysis in a randomized clinical trial. *JAMA Netw Open*. 2018;1(2):e180334. doi:10.1001/jamanetworkopen.2018.0334

eTable. Single Mediatory Pathways With Unstandardized Values

eFigure 1. Full Multivariate Model With Unstandardized Values

eFigure 2. Parsimonious Multivariate Path Model With Unstandardized Values

This supplementary material has been provided by the authors to give readers additional information about their work.

© 2018 Silverstein M et al. *JAMA Network Open.*

Appendix – Unstandardized Values

The following Tables/Figures present the same data as in the main manuscript, but with unstandardized values.

eTable: Single Mediatory Pathways With Unstandardized Values (Analogous to Table 2 in the main paper)

	Relationship between PSE and Mediators		Relationship between Mediators and Outcomes	
	Path coefficient for change score associated with participation in PSE (95% CI)	Standardized path coefficient for change score associated with participation in PSE (95% CI)*	Rate ratio of depression elevations per 1 unit difference of change in mediator score (95% CI)	Rate ratio of depression elevations per standard deviation difference of change in mediator score (95% CI)*
Overall Problem Solving ability	0.17 (-0.13, 0.47)	0.08 (-0.07, 0.23)	0.89 (0.84, 0.95)	0.80 (0.71, 0.90)
Mastery	-0.13 (-0.45, 0.19)	-0.05 (-0.19, 0.09)	1.06 (1.01, 1.11)	1.14 (1.01, 1.29)
Self-Esteem	0.46 (-0.13, 1.05)	0.11 (-0.03, 0.25)	0.94 (0.91, 0.98)	0.79 (0.69, 0.91)
Social coping	0.10 (-0.03, 0.23)	0.12 (-0.03, 0.27)	1.02 (0.89, 1.17)	1.02 (0.90, 1.15)
Behavioral Activation	3.10 (0.11, 6.09)	0.15 (0.01, 0.30)	0.99 (0.98, 0.99)	0.74 (0.65, 0.83)
Avoidant coping‡	-0.02 (-0.12, 0.08)	-0.02 (-0.17, 0.13)	1.20 (1.01, 1.44)	1.14 (1.01, 1.30)
Problem-focused coping	0.12 (0.02, 0.22)	0.17 (0.03, 0.31)	1.05 (0.89, 1.24)	1.04 (0.92, 1.17)
Perceived Stress‡	-1.45 (-2.53, -0.37)	-0.11 (-0.19, -0.03)	0.93 (0.92, 0.95)	0.38 (0.30, 0.47)

*Coefficients represent percentage of a standard deviation in change of a mediator score.

‡Negative values for change indicate improvement.

© 2018 Silverstein M et al. *JAMA Network Open.*

eFigure 1. Full Multivariate Model With Unstandardized Values (Analogous to Figure 2 in the main paper)

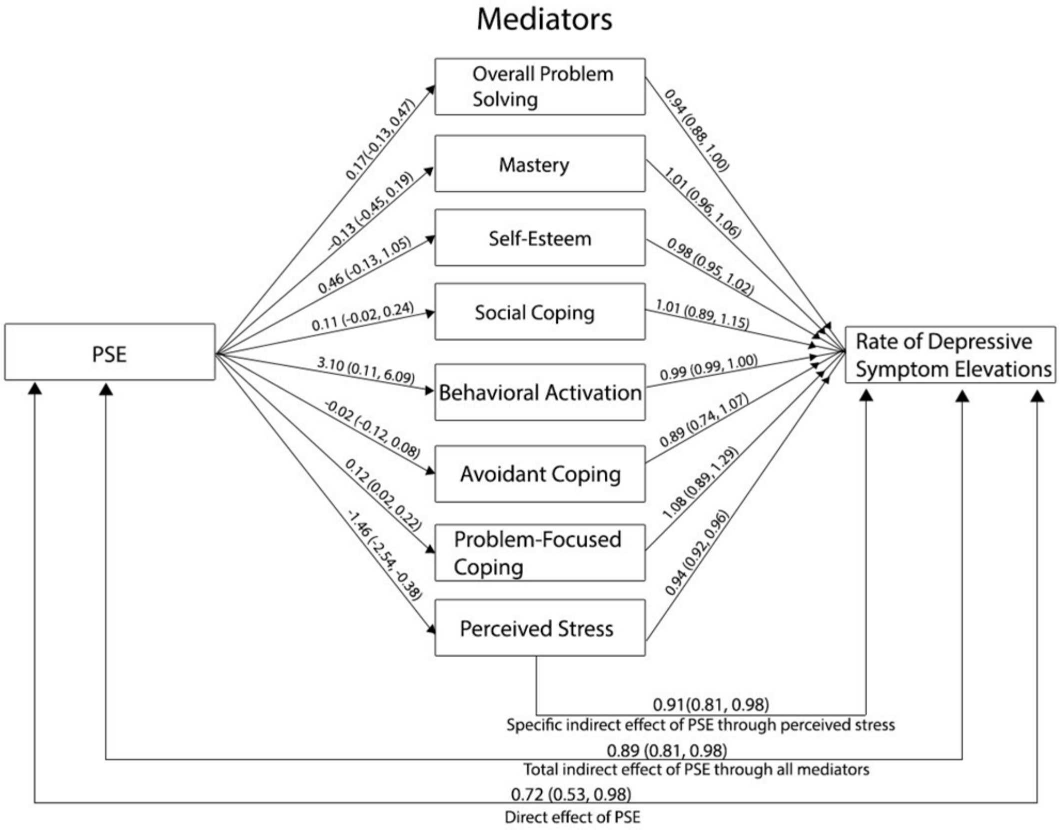

© 2018 Silverstein M et al. *JAMA Network Open.*

eFigure 2 Parsimonious Multivariate Path Model With Unstandardized Values (Analogous to Figure 3 in the main paper)

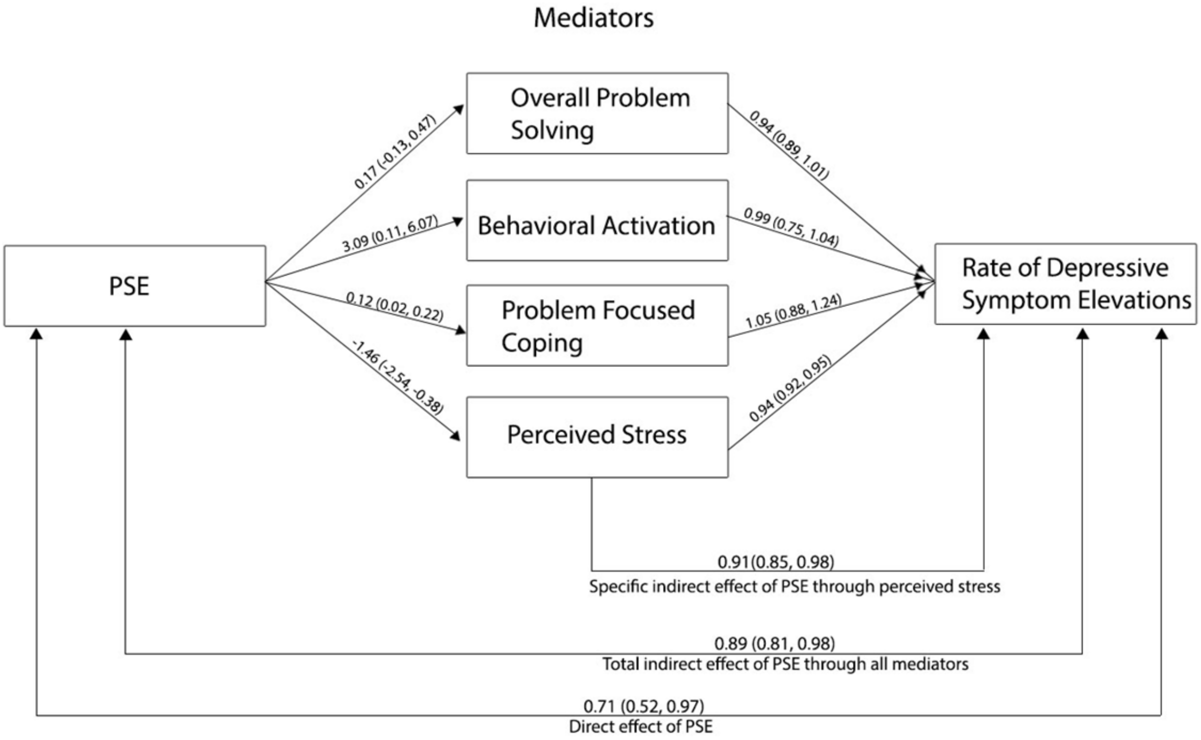

© 2018 Silverstein M et al. *JAMA Network Open.*
